# Partners’ experiences of induction of labour in inpatient and outpatient settings: a questionnaire-based qualitative study within the Swedish OutPatienT InductiON (OPTION) trial

**DOI:** 10.1136/bmjopen-2026-124576

**Published:** 2026-11-03

**Authors:** Camilla Buskhe Pauli, Marianne Wadman, Helen Elden, Ylva Carlsson, Anna Wessberg, Verena Sengpiel

**Affiliations:** 1 Department of Obstetrics and Gynecology, Sahlgrenska University Hospital, Gothenburg, Region Västra Götaland, Sweden, Gothenburg, Sweden; 2 Department of Obstetrics and Gynaecology, Institute of Clinical Sciences, Sahlgrenska Academy, University of Gothenburg, Gothenburg, Sweden; 3 Department of Obstetrics and Gynaecology, Halland Hospital Varberg, Region Halland, Varberg, Sweden; 4 Institute of Health and Care Sciences, Sahlgrenska Academy, University of Gothenburg, Gothenburg, Sweden; 5 Centre of Perinatal Medicine and Health, Gothenburg, Sweden, Institute of Clinical Sciences, Sahlgrenska Academy, University of Gothenburg, Gothenburg, Sweden

**Keywords:** Life Change Events, Social Support, Midwifery, OBSTETRICS

## Abstract

**Objective:**

With increasing induction of labour (IOL) rates, many partners experience IOL. Despite them often being the main support person, knowledge of partners’ IOL experiences remains limited. This study aimed to describe partners’ IOL experiences in both inpatient and outpatient settings.

**Design:**

A qualitative study based on partners’ free-text answers in two electronic questionnaires answered at the beginning of IOL and 3 months postpartum between 1 December 2021 and 24 August 2024. The answers were analysed using qualitative content analysis.

**Setting:**

The study is part of the Swedish randomised multicentre OutPatienT InductiON of labour (OPTION) trial with 31 out of 45 Swedish maternity hospitals participating.

**Participants:**

Partners of women enrolled in OPTION trial, aged >18 years, were invited to join the questionnaire part of the trial. Of 993 participating partners, 487 partners answered any of the free-text questions.

**Results:**

The analysis resulted in four categories with eight subcategories. In the first category, *Taking a leap into the unknown*, partners described entering unfamiliar territory where the experience was shaped by their pre-understandings and expectations. The second category, *Being at the mercy of the setting and available resources*, reflects how the IOL setting and hospital organisation shaped the experience. The third category, *Wanting to support the partner while not feeling in control*, captures the emotional roller coaster of navigating an unfamiliar situation while consistently prioritising the needs of the pregnant partner. Finally, *Finding oneself in need of support* highlights partners’ need to preserve their own energy and receive professional support from healthcare staff to be able to support the woman.

**Conclusion:**

Partners’ IOL experiences varied widely. Clear information, structured organisation, professional support, and a comfortable care environment helped partners feel better equipped to support the woman undergoing IOL. These modifiable aspects of care may improve the overall IOL experience for both the woman and accompanying partner.

**STRENGTHS AND LIMITATION OF THIS STUDY**

- The multicentre design and inclusion of partners with varying characteristics and experiences from both inpatient and outpatient induction of labour provided breadth to the dataset.
- Rigour was strengthened through an iterative analytical process and regular discussions among authors from different obstetric professions, enhancing dependability and confirmability.
- The pseudo-anonymous format may have facilitated open and honest descriptions of experiences.
- Use of written free-text responses limited opportunities for follow-up questions and deeper exploration of participants’ experiences and views.
- Findings should be interpreted in the context that participants were partners of women who consented to participate in the outpatient induction trial and were open to outpatient induction of labour, which may affect generalisability.

## Introduction

 In Sweden, as in many high-income countries, most women choose their partner as primary support person during labour and birth.^1 2^ The birth of one’s child is a major life event for both parents. Partners are expected to support the pregnant woman through pregnancy and birth,^1 3 4^ but often feel that they lack experience and knowledge to do so.^5^ Adequate support during labour and birth improves medical outcomes of women and their children,^6^ and a good birth experience facilitates transition to parenthood for both parents.^4^

With induction of labour (IOL) rates increasing to approximately 30% in high-income countries,^7–9^ many partners share the IOL experience. Like in other countries, increasing IOL rates in Sweden are mainly caused by new national guidelines regarding prolonged pregnancy.^10 11^ Generally, women induced due to prolonged pregnancy are healthy and have experienced uncomplicated pregnancies. For low-risk IOLs, outpatient IOL has been introduced in several countries. Outpatient IOL typically involves initiation IOL at the hospital, followed by the woman returning home during cervical ripening before being re-assessed or admitted when labour progresses or further intervention is required.^12 13^ In Sweden, the safety, efficacy, experience and health economics of low-risk outpatient IOL is currently investigated in the Swedish randomised multicentre OutPatienT InductiON (OPTION) trial.^14^

Despite partners often being the primary support person during the phase of cervical ripening, and especially in case of outpatient IOL, there is hardly any research on partners’ experiences of IOL—regardless of inpatient or outpatient IOL. In a recent study from the UK, couples were interviewed about their experiences of outpatient IOL. Partners expressed a need for clear and comprehensive information and emphasised that the pregnant woman should be able to choose the setting, although some partners preferred the outpatient setting themselves.^15^

The OPTION trial^14^ provides a unique opportunity to investigate partners’ experiences of both inpatient and outpatient IOL. Such knowledge is important for informing how IOL care can be organised to better support both women and their partners.

The aim of this study was to describe partners’ experiences of IOL in both inpatient and outpatient settings.

## Methods

### Study design and setting

This study was based on qualitative inductive content analysis of written free-text answers collected through two electronic questionnaires administered within the OPTION trial.^16^

The method is suitable when little is known about a phenomenon and is widely used for the systematic analysis of lived experiences based on textual data, such as transcribed interviews or written responses.^16^

In Sweden, maternity and delivery care is publicly funded and partners are encouraged to accompany the pregnant woman. The study is part of the Swedish multicentre OPTION trial (EU CT no: 2023-507164-3900) where a total of 31 out of 45 hospitals participated.^14^ Women planned for low-risk IOL, for example, a full-term pregnancy with a singleton foetus in cephalic presentation without risk factors requiring continuous maternal or fetal monitoring, aged >18 years, and who could communicate with the hospital by phone, were eligible for participation. After induction with either a balloon catheter or oral prostaglandins, women were randomised to continue IOL at the hospital or to return home until the active phase of birth started or complications arose. Couples randomised to the outpatient setting could contact the hospital and talk to a midwife at any time and return to the hospital whenever they wanted to. All women in the outpatient group had a planned appointment with new cervix examination, fetal monitoring and plan for continued IOL care at the hospital after 24 hours. If there was need for further cervical ripening, the woman and her partner could return home for another 24 hours before final admission to the hospital. All births were planned to take place in the hospital.^14^

### Recruitment and participants

Partners of women participating in the OPTION trial were invited to join the questionnaire part of the study at the time of the woman’s inclusion in the trial. Partners participating in the OPTION trial between 1 December 2021 and 24 August 2024 were eligible for the present study. All participating partners provided written informed consent before completing the first questionnaire.

### Data collection

The study is based on free-text answers from electronic questionnaires completed by partners participating in the OPTION trial.^14^ All partners received study-specific questionnaires administered at two timepoints; at inclusion before randomisation and 3 months postpartum. The questionnaires included both fixed-response items and opportunities to provide free-text comments regarding experiences of overall experiences of the IOL process (table 1).^14^ The present study is based on the free-text responses. In a substudy at the largest site, participating partners were asked an additional set of free-text questions as part of the 3 months postpartum questionnaire (table 1). Questionnaires were provided in Swedish and English. Information on demographics was obtained from the Swedish Pregnancy Register on group level to maintain blinding in the OPTION trial. Responses were pseudonymised, meaning that participants were assigned a study ID and direct identifiers were removed prior to analysis.

**Table 1:** Free-text questions in the electronic outpatient induction trial questionnaires

Time point	Questions
At inclusion	Is there anything else you would like to share with us in relation to the induction of your/your partner’s labour?
Three months postpartum to all participants	What is your experience of your partner’s induction of labour? Is there something else that you want to share with us in relation to the induction of labour?
Additional questions 3 months postpartum to participants from the largest site	How did you feel when the induction of your partner’s labour began? What support did you receive from the hospital staff during your partner’s labour induction? What support would you have liked to receive? How much control did you feel that you had during the induction of your partner’s labour and what was it that made you feel that you had control?

### Data analysis

The analysis followed the preparation, organisation and reporting phases described by Elo and Kyngäs.^16^ During the preparation phase, all responses were read repeatedly to gain familiarity with the data. All free-text responses from the two questionnaire time points were analysed together as one dataset. Responses submitted in English were analysed alongside Swedish responses without translation during coding. Meaning units relevant to the study aim were identified and highlighted. These meaning units were compared and discussed in relation to the study aim. The material was subsequently coded independently by two of the authors, and any differences were discussed until agreement was reached. Codes with similar content were grouped into subcategories and categories. The analysis was conducted iteratively, moving back and forth between the full dataset and the emerging subcategories and categories to ensure that no information was lost. Through iterative discussion and constant comparison with the original responses, subcategories were further developed into broader categories representing the latent content of the data. Disagreements regarding interpretation were resolved through discussion until consensus was reached. The subcategories and categories were continuously discussed and refined by all authors until consensus was achieved. NVivo (Lumivero) software was used to facilitate the analysis.^17^ The authors consisted of midwives and obstetricians with experience of maternity and childbirth care and both inpatient and outpatient IOL care. The authors were aware that their clinical experience could influence data interpretation. To promote reflexivity, coding and interpretation were conducted independently before being discussed within the multidisciplinary research team. The study followed the Standards for Reporting Qualitative Research (SRQR).^18^

## Result

Of the 993 partners participating in the OPTION questionnaire part, 487 (49%) answered at least one free-text question and were included in this study. Among these were 63 partners (13% of the 487) recruited at the largest site receiving the more detailed postpartum questions (see table 1). Participants were 19–57 years of age, and the majority accompanied a woman expecting her first child (table 2a–b). The free-text answers ranged from brief comments to detailed narratives describing experiences and reflections on the IOL process, providing both breadth and depth relevant to the study aim. Experiences from both inpatient and outpatient IOL contributed to all categories. Rather than representing two distinct experiences, partners’ accounts suggested that factors such as access to information, staff support, waiting times, opportunities for rest and the overall organisation of care were more influential than the setting itself.

**Table 2:** Partner characteristics and women’s birth-related data

(a) Partner characteristics		All	Answered free text
Partner age		33.1 (5.0)	33.4 (4.9)
Partner attended parenting support	Yes	440 (44.3%)	232 (47.6%)
	No	454 (45.7%)	212 (43.5%)
	Missing	99 (10%)	43 (8.8%)
Partner gender	Female	14 (1.4%)	7 (1.4%)
	Male	976 (98.3%)	479 (98.4%)
	Unknown	3 (0.3%)	1 (0.2%)
**(b) Women’s birth-related data**		**All**	**Answered free text**
Randomisation group	Outpatient	480 (48.3%)	252 (51.7%)
	Inpatient	466 (46.9%)	231 (47.4%)
	Not randomised*	47 (4.7%)	4 (0.8%)
Hospital type	University hospital	559 (56.3%)	289 (59.3%)
	Not university hospital	434 (43.7%)	198 (40.7%)
Mode of birth	Spontaneous vaginal birth	703 (70.8%)	348 (71.5%)
	Instrumental vaginal birth	128 (12.9%)	60 (12.3%)
	Acute caesarean section	162 (16.3%)	79 (16.2%)
Parity	Nulliparous	799 (80.5%)	395 (81.1%)
	Multiparous	194 (19.5%)	92 (18.9%)
Total bleeding	>1000 mL	131 (13.2%)	60 (12.3%)
	Missing	<5 (0.5%)	<5 (1%)
Third-degree or fourth-degree tear	Yes	37 (3.7%)	17 (3.5%)
Neonatal resuscitation†	Yes	29 (2.9%)	17 (3.5%)

Values are presented as n (%) or mean (SD).

* Women are included in the OPTION trial before initiation of induction of labour and monitored at the hospital for 45 min before randomisation. Some women might experience exclusion criteria such as start of labour or complications such as bleeding and thus will be excluded before randomisation. As questionnaire 1 is answered before randomisation, some women to participating partners in this study will not have been randomised.

† Neonatal resuscitation was defined as any use of mask ventilation, intubation or cardiac massage.

OPTION, OutPatienT InductiON trial.

Four categories with eight subcategories describing partners’ experiences of IOL emerged from the analysis: *Taking a leap into the unknown*, *Being at the mercy of the setting and available resources*, *Wanting to support the partner while not feeling in control* and *Finding oneself in need of support* (table 3). Partners’ pre-understandings, expectations and preconceptions impacted their IOL experience. They described feeling unprepared, uncertain about what to expect and that their actual IOL experience often was better or worse than anticipated. Partners described how their feelings of safety, satisfaction and comfort depended on the setting and the hospital’s resources and organisation. IOL awakened a variety of emotions, varying from joy and excitement to worry, anxiety and nervousness. Partners described feeling that they had little or no control during the IOL process but also reported accepting and even expecting this lack of control. Partners consistently prioritised the pregnant partner’s physical and emotional needs over their own, often suppressing or setting aside their own worries, uncertainty and distress in an effort to provide support throughout the IOL process. At the same time, partners described their own need of care from the staff welcoming information, guidance, emotional support and opportunities to rest.

**Table 3:** Categories and subcategories describing partners’ experiences of induction of labour in inpatient and outpatient settings

Category	Subcategory
Taking a leap into the unknown	To start the induction of labour process evokes emotions Expectations and prior knowledge shape the experiences in unknown territory
Being at the mercy of the setting and available resources	The setting should provide comfort The hospital’s organisation is important to feel safe
Wanting to support the partner while not feeling in control	Participating without being in control Standing back to support the woman
Finding oneself in need of support	Receiving continuous information and support is vital to cope Needing recovery and rest to handle induction of labour and birth

### Taking a leap into the unknown

Partners entered the IOL process with different expectations, previous experiences and needs for reassurance, which shaped how they experienced IOL.

### To start the induction of labour process evokes emotions

At the beginning of the IOL process, partners described a wide range of emotions, ranging from stress and nervousness to calmness and anticipation. The concept of IOL evoked curiosity in some and scepticism in others, resulting in feelings of either excitement or worry, and often a combination of both. Some partners experienced the process as nerve-wracking and perceived the use of medication to induce labour as unnatural and unpleasant. Others expressed positive emotions, such as relief that the pregnancy was nearing its end, happiness and excitement about progressing towards active labour without having to wait for spontaneous labour onset.

I am very positive about induction, as I have seen a significant change in my partner’s well-being from simply waiting aimlessly at home for labour to start naturally. (Partner of nulliparous woman, Questionnaire 1, at inclusion)

### Expectations and prior knowledge shape experiences in unknown territory

Partners described that the IOL experience did not unfold as they had expected. This discrepancy was largely attributed to their prior knowledge and pre-understandings of IOL. Their expectations did not always align with the actual course of events, and IOL was experienced as both easier and more challenging than anticipated. Some described the IOL process as ‘not knowing what they were in for’ and as ‘stepping into unknown territory.’ Initiating the IOL process was also described as being shaken out of one’s role, as partners had not prepared to act as support person in the context of IOL. Preparations for spontaneous labour did not always apply to IOL, contributing to uncertainty and a sense of being lost.

The part of calming my partner, calling the maternity ward, giving massages, and being a source of support, in a way that part fell away. It is still a significant part of what I had prepared for, so I think that contributed to me being somewhat unsettled in my role. (Partner of nulliparous woman, Questionnaire 2, at 3 months postpartum)

### Being at the mercy of the setting and the available resources

Feelings of safety and comfort during IOL were linked to available resources. Regardless of the setting, partners described hospital resources, the physical environment and the accessibility of staff integral to the IOL experience. There were many accounts of constantly waiting for the next step of IOL. Partners reported frustration when delays in staff availability led to a sense of being stuck and not progressing in the IOL process.

### The setting should provide comfort

The physical environment was an important factor for partners and could enhance their IOL experience. Some partners undergoing inpatient IOL expressed satisfaction with remaining in the hospital throughout the process. A stimulating environment was perceived as beneficial. Many described the IOL as prolonged and tedious, emphasising that a setting providing possibility to pass time during the early stages was important in helping them cope. In retrospect, some partners who experienced inpatient IOL indicated that they would have preferred to start the process at home. Partners reported disappointment with boring hospital rooms and uncomfortable beds. Others described exhaustion following a long and intense IOL, often involving several days in the hospital with little to no sleep before the onset of active labour. Partners who began the IOL process at home reported positive experiences, stating that being in a familiar environment helped both them and the pregnant woman to relax and normalise the process. They experienced a greater sense of autonomy, and some expressed gratitude for being able to leave the hospital during the early stages of IOL, noting discomfort in hospital settings in general. The ability to maintain everyday routines was also described as positive, as it made the IOL process feel more manageable.

We stayed at home during that time, and we both felt that it was really good and comfortable to be in our own home. We could live as usual and do whatever we felt like in the meantime. (Partner of multiparous woman, Questionnaire 2, at 3 months postpartum)

### The hospitals’ organisation is important to feel safe

Regardless of inpatient or outpatient setting, partners felt dependent on the hospital’s resources for care. Many described frustrations with waiting times between different stages and interventions during IOL, perceiving the process as prolonged and drawn out as a result. A lack of resources and limited staff availability were seen as negatively affecting their experience. In contrast, some partners described staff as consistently present, informative and supportive, which contributed to a positive IOL experience. Others expressed disappointment when staff appeared stressed and insufficiently responsive to the pregnant woman’s need for attention and pain relief. The ability to communicate with healthcare professionals at any point during IOL was considered important. Having access to a midwife at any time contributed to a sense of safety for those at home. Partners in outpatient settings also emphasised the importance of having a reserved hospital room and feeling secure at home, knowing that they could return to the hospital and be readmitted at any time.

It felt good to be able to stay at home, even though we don’t have our own car. If we hadn’t known that we would have a secure place when we came in, we would never have dared to leave, since we don’t have a car. (Partner of nulliparous woman, Questionnaire 2, at 3 month*s* postpartum)

### Wanting to support the partner while not feeling in control

Following the woman’s physical birthing process was experienced as being beyond the partners’ control. Although this lack of control was described as a natural and accepted part of the process, it could still be challenging. The IOL process evoked a wide range of emotions, which varied considerably among partners.

### Participating without being in control

Partners described having to watch their pregnant partner being in pain while feeling powerless themselves. Others described not needing a sense of control, attributing this to receiving adequate information during the IOL process, which helped coping and appreciating the experience. The inability to control the situation or the IOL process was described as ranging from something natural and expected to something challenging and stressful.

During the natural process, you have no control over the contractions and so on, but with a medical induction I felt that the control was there, just not with me and my partner, which was a disarming experience. (Partner of nulliparous woman, Questionnaire 2, at 3 months postpartum)

### Standing back to support the woman

Partners described experiencing a wide range of intense emotions during IOL, yet their accounts often emphasised the necessity of putting their own needs aside to prioritise the woman’s well-being. Their reflections highlighted how their personal feelings were often secondary to their supportive role. Whether the process unfolded quickly or slowly, calmly or with difficulty, partners focused on adapting themselves to meet the woman’s needs. The woman’s pain, fatigue and the duration of labour strongly shaped not only the partners’ experiences but also their sense of responsibility to remain steady and available. Partners described suppressing their own discomfort, fear or uncertainty in order to provide continuous emotional and practical support. Feelings of inadequacy or unease were often managed internally, as attention was directed towards ensuring the woman felt supported. Overall, the experience of IOL for partners involved not only navigating complexity and uncertainty but also consciously subordinating their own emotional needs to fulfil their role as a source of stability and care.

For a long time, she never got a break. Very intense, even for me, as I did everything I could to support and calm her. Without much success. (Partner of nulliparous woman, Questionnaire 2, at 3 months postpartum)

### Finding oneself in need of support

Partners had their own need for care and support during IOL. They wanted to be good support persons, maintain sufficient energy and receive continuous information and professional support from the staff. Partners felt that they needed support themselves in order to support the woman giving birth.

### Receiving continuous information and support is vital to cope

Receiving emotional support from the staff and having the opportunity to continuously ask questions contributed to a positive IOL experience. Partners mostly praised the hospital staff, their emotional support and their care for the pregnant woman. They were pleased and sometimes surprised by the care and support they received as partners. Receiving information tailored to the couple was important.

I felt that it was just as acceptable for me to ask if I was unsure about something as it was for the birthing person to do so. That was a great source of reassurance. (Partner of multiparous woman, Questionnaire 2, at 3 months postpartum)

Some partners described receiving adequate information at the beginning of IOL, but when the process stagnated and did not progress as expected, information became insufficient, and a clear plan was lacking. Some felt ignored, which contributed to feelings of uncertainty. Some described it as frightening to be the main support person and expressed a wish that staff provided more guidance on how to act.

### Needing recovery and rest to handle induction of labour and birth

Partners described feeling extremely tired during IOL. The lack of energy affected their ability to cope, and they reflected on how they wished they had taken more opportunities to rest in the early stages of IOL. A long IOL process could be experienced as extremely draining, and this negatively affected the experience of subsequent active labour and birth.

It became very prolonged as the induction took several days and we had to wait for a place at the delivery ward. I would much rather have waited at home or had a bed so I wouldn’t have had to sit upright so much. (Partner of nulliparous woman, Questionnaire 2, at 3 months postpartum)

Partners expressed a need for their own physical well-being, including rest and recovery, to be supported in order handle the at times unpredictable situation. For some, feeling uncomfortable or tired was considered part of the experience, while others described how being well-rested helped them cope with challenging circumstances.

## Discussion

To our knowledge, this is one of the first studies to examine partners’ experiences of IOL across both inpatient and outpatient settings. Partners wanted circumstances that allowed them to be the best support person they could be. Being the partner of a woman experiencing IOL can be challenging, as many aspects are beyond the partner’s control. Our findings indicate that partners had limited knowledge of the IOL and birth processes and expressed a need for continuous information throughout the process. Acting as a support person often meant being dependent on the hospital’s resources and the organisation of care.

Strengths of this study include the large number of partners who provided free-text responses, the multicentre setting with recruitment from all over Sweden, and the inclusion of experiences from both inpatient and outpatient IOL. The inclusion of partners of both nulliparous and multiparous women, from all over Sweden, giving birth at university as well as minor hospitals, may support the relevance of the findings across similar maternity care contexts. Due to the nature of data collection, recruitment was not guided by saturation. Instead, we considered the large number of participants, the variation in responses, and the breadth of experiences represented in the dataset to provide sufficient information to address the study aim. However, transferability should be considered in light of the study population and design. Limitations include the use of written free-text responses, which did not allow for follow-up questions. As questionnaires were completed 3 months postpartum, participants may have reflected on the IOL process considering the birth experience and outcomes. Consequently, some reported experiences may not exclusively relate to the IOL process itself. When interpreting the findings, it should be noted that participants were recruited through the OPTION trial, and women who chose to participate may have been more favourably disposed towards outpatient IOL than the general population. However, our findings also included several accounts from partners who—themselves—preferred an inpatient setting. This suggests that perspectives on the preferred care setting varied among participants.

As only 49% of participating partners provided free-text responses, the experiences reported may not fully reflect those of all partners participating in the OPTION trial. However, the use of questionnaire-based free-text responses enabled participation from a substantially larger and geographically more diverse sample than would have been feasible in an interview study. While not all OPTION participants provided free-text answers, partners providing free text answers were similar to the group of all participants regarding age, parity, mode of birth, and attendance at parenting support. Dependability and confirmability were strengthened through an iterative analytical process and repeated discussions among authors from different obstetric professions (midwives, medical students, obstetricians). When interpreting the findings, it needs to be considered that participants were partners of women who participated in the OPTION trial and thus were open to outpatient IOL.

Participants experienced a variation of strong emotions in relation to IOL, comparable to those reported by their partners undergoing IOL.^19 20^ Involvement in decision-making, opportunities for distraction, adequate care and sufficient information were crucial for a good experience, consistent with previous studies on first-time fathers during labour and childbirth.^21 22^ Some partners reported a lack of information and support, while positive experiences were linked to well-organised and efficient organisation. These findings align with previous research by Yuill *et al*,^15^ although the UK study does not clearly distinguish between partners’ and women’s experiences. Although research in this field is limited, our findings are consistent with studies from other healthcare contexts where next of kin accompany patients. In the category *Taking a leap into the unknown*, partners emphasised the importance of preparation and mindset prior to IOL. Previous research suggests that targeted information for next of kin can improve their ability to support the woman during childbirth and other hospital procedures.^23–25^ These findings indicate a potential need for educational materials tailored to partners undergoing IOL.

In line with earlier studies, involvement, choice and information were central to a positive IOL experience.^21 26 27^ In *Being at the mercy of the setting and available resources*, partners described how the care setting and access to staff influenced their experience. Similar findings have been reported in studies of next of kin in hospital-at-home, intensive care and childbirth settings, where access to rest, distractions and a safe or homelike environment were considered supportive.^28–30^ In the category *Wanting to support the partner while not feeling in control*, partners described the challenge of managing emotional variability during IOL, echoing findings from studies on active labour.^1 21 26 27^ Additionally, partners have the natural role of primary support person during early stages of labour but can also feel minimised in their role and be more focused on practical tasks.^31^ Finally, in the category of *Finding Oneself in Need of Support*, partners expressed a need for information and support. This finding is consistent with previous studies reporting partners’ need for support during pregnancy, birth and postpartum.^32 33^ During hospital stays, when a next of kin is expected to remain with and support the patient, previous research has shown that support from healthcare staff helps the next of kin cope with the situation.^33 34^

For some partners, IOL involved substantial emotional and practical demands, who are expected to provide continuous support while navigating one of the most emotionally demanding experiences of their own lives. These findings highlight the need for healthcare providers to recognise partners as individuals with their own support needs and to actively evaluate how care settings, organisation and resources impact their ability to cope and provide support. Individualised information, structured care pathways and supportive environments were described as helping partners feel better supported during IOL. Future research should further explore how different models of care can be optimised to reduce partner strain and identify targeted interventions that support both partners’ well-being and their capacity to support the woman during IOL.

## Conclusion

Partners’ experiences of IOL varied widely and were shaped by the organisation of care, information, professional support and the care setting. Structured and accessible care helped partners feel safe and enabled them to support the woman undergoing IOL and giving birth. Partners welcomed an environment supporting autonomy and rest. The option to spend part of the IOL process at home was perceived positively by some partners.

Further research is needed to identify how partners can best be prepared and supported throughout IOL in both inpatient and outpatient settings to help them support their partner and improve IOL experiences for both pregnant women and their partners.

## Data Availability

No data are available.
