# Supplementary material for: Partners’ experiences of induction of labour in inpatient and outpatient settings: a questionnaire-based qualitative study within the Swedish OutPatienT InductiON (OPTION) trial
Source: BMJ Open. 2026 Nov 3;16(11):e124576. doi: 10.1136/bmjopen-2026-124576 (PMC13735268; doi:10.1136/bmjopen-2026-124576)
Supplement: Supplementary file 1 [file bmjopen-2026-124576.reviewer_comments.pdf]

PEER REVIEW HISTORY

BMJ Open publishes all reviews undertaken for accepted manuscripts. Reviewers are asked to complete a checklist review form and are provided with free text boxes to elaborate on their assessment. These free text comments are reproduced below.

ARTICLE DETAILS

Title (Provisional)

Partners´ experiences of induction of labour: A questionnaire-based qualitative study within the Swedish Outpatient Induction Trial (OPTION)

Authors

Buskhe Pauli, Camilla; Wadman, Marianne; Elden, Helen; Carlsson, Ylva; Wessberg, Anna; Sengpiel, Verena

---

VERSION 1 - REVIEW

**Reviewer**	**1**
**Name**	**Karataşlı, Volkan**
**Affiliation**	**Balikesir Atat□□rk City Hospital**
**Date**	**13-Aug-2026**
**COI**	**None**

The manuscript, "Partners' experiences of induction of labour: A questionnaire-based qualitative study within the Swedish Outpatient Induction Trial (OPTION)," addresses an important and relatively understudied aspect of maternity care. The partner is often expected to act as the primary support person during induction of labour (IOL), yet the partner's own experiences and support needs receive considerably less attention. The multicentre context, inclusion of both inpatient and outpatient IOL, relatively large number of free-text respondents, and use of qualitative content analysis are notable strengths. The four categories are clinically recognizable and potentially useful for improving IOL care. However, several methodological and reporting issues require clarification, particularly regarding the distinction between inpatient and outpatient experiences, heterogeneity of data collection, participant selection, and qualitative rigor.

1. The stated focus on inpatient versus outpatient IOL is not sufficiently reflected in the analysis.

2. The data collection strategy differs substantially between participants and may have influenced the findings.

3. Baseline and postpartum responses represent conceptually different types of data.

4. The 49% free-text response rate introduces important self-selection bias.

5. Participant characteristics are insufficiently reported for assessing transferability.

6. The distinction between characteristics of the partner and characteristics of the pregnant woman should be improved.

7. The potential influence of birth outcome and complications on the reported IOL experience deserves greater consideration.

8. The analytic procedure needs greater methodological detail.

9. Researcher reflexivity is insufficient despite being indicated in the SRQR checklist.

10. The authors should clarify the methodological position regarding saturation or information sufficiency.

11. Data richness should be described.

12. The wording regarding credibility and transferability is stronger than the available participant data support.

13. The findings related to outpatient IOL should be interpreted in the context of trial selection.

14. The manuscript should more clearly distinguish findings from clinical recommendations.

15. The term “pseudo-anonymous” requires clarification.

16. Minor spelling errors should be corrected.

**Reviewer**	**2**
**Name**	**Cheyne, Helen**
**Affiliation**	**University of Stirling, NMAHP Research Unit**
**Date**	**14-Aug-2026**
**COI**	**None**

Thank you for inviting me to review this interesting article. The study aims to describe the experiences of partners of women undergoing labour induction, both in and outpatient, by analysing the free text data on online questionnaires. This is an interesting topic that is highly relevant currently as rates of labour induction rise in many countries. The experiences of partners are important but often overlooked and under researched.

Overall, this is a well written paper and enjoyable to read. I have a few specific comments and suggestions for minor changes. These mostly relate to the methods section which I felt could be more logically structured, with some more detail for clarity.

Introduction

Line 127 For low-risk IOLs, outpatient IOL has been introduced in several countries. I suggest adding a sentence to explain outpatient IOL.

Despite partners often being the primary support person during the phase of cervical ripening – through most of this paper no distinction is made between cervical ripening and the later phases of the IOL process. Do you intend this paper to make that distinction? This may be important as you also have a focus on outpatient – which generally relates only to the process of cervical ripening. If so, I think you need to thread the differences between cervical ripening and IOL throughout.

The first two sentences of the concluding paragraph in the introduction repeat what is written in the paragraph above, I suggest removing the repetition.

Methods

Design – the sentence justifying the design doesn't really explain the design. I suggest adding an additional sentence in this section to clarify that this was a qualitative analysis of free text responses within online questionnaires. The current sentence justifying the method – as required by the checklist – is justifying content analysis not the questionnaire / free text comments approach that was actually the design of the study. I think this could be clearer.

Line 155 onwards describing the OPTIONS Trial. The sentence Partners to participating women were invited to join the questionnaire part of the study. This does not fit well here. It does not give enough information about the partners participation, and it breaks up the description of the trial. I suggest completing the description of the Trial and then go on to describe the questionnaires more fully.

Line 162 – you say - women were 163 randomized to continue IOL at the hospital, or to return home until the active phase of birth started – at few sentences later you say they were scheduled to return after 24 hours for assessment.

Following completion of the description of the OPTIONS Trial, I suggest you go on to provide more detail on the questionnaires. For example, were the questionnaires just for partners or were they for the women but with some questions for partners? What were the questionnaires about?

Recruitment – what was the procedure for recruitment of the partners and how did they provide written consent – for example, was their consent gained at the start of the electronic questionnaire or were they recruited at the same time as the women were initially recruited to the trial?

Data collection – why were some partners given additional free text questions? Can you clarify if there were two or three questionnaires? The abstract says two but the first sentence in the Data Collection section suggests three – inclusion, randomisation and postpartum.

Data analysis

This section is quite well described, please clarify if data for all questionnaire time points were analysed as one data set? If so, I wondered about the usefulness of having two or three questionnaires. Were any questionnaires returned in English and if so, how was data translation handled.

How many of the responses were from partners in the home IOL arm of the study?

Is there any more information available on the participants characteristics? for example, were all male? You claim that a strength of the study is the variation in participants but don't include much information to back that up.

Findings

The findings are generally well written and interesting. I likeed the summary of findings at the start but then going into theme one it seems a little repetitive with the first paragraph just repeating the summary above.

Is it possible to add some identifiers to the quotes? At present most of the quotes could have come from just one or two partners. Did most of the data come from the larger unit where more questions were asked?

Discussion

You say - Variation in participant characteristics and hospital settings strengthens the credibility and potential transferability of the findings. Yet you only include age and attendance at classes for partner variation and no data on hospital variation.

VERSION 1 - AUTHOR RESPONSE

Reviewer: 1
Dr. Volkan Karataşlı, Balikesir Atat��rk City Hospital
Comments to the Author:
The manuscript, "Partners' experiences of induction of labour: A questionnaire-based qualitative study within the Swedish Outpatient Induction Trial (OPTION)," addresses an important and relatively understudied aspect of maternity care. The partner is often expected to act as the primary support person during induction of labour (IOL), yet the partner's own experiences and support needs receive considerably less attention. The multicentre context, inclusion of both inpatient and outpatient IOL, relatively large number of free-text respondents, and use of qualitative content analysis are notable strengths. The four categories are clinically recognizable and potentially useful for improving IOL care. However, several methodological and reporting issues require clarification, particularly regarding the distinction between inpatient and outpatient experiences, heterogeneity of data collection, participant selection, and qualitative rigor.

Thank you very much for taking the time to review our manuscript and providing valuable feedback.

1. The stated focus on inpatient versus outpatient IOL is not sufficiently reflected in the analysis.

We apologise that our original wording may have suggested that the aim was to compare the two settings. The purpose of the study was instead to provide insights into experiences from both settings. Our findings indicate that both settings have distinct benefits and limitations, and that the perceived advantages and disadvantages vary between individuals. To clarify this, we have revised the Results section accordingly.
Page 9, line 225-229: "Experiences from both in- and outpatient IOL contributed to all categories. Rather than representing two distinct experiences, partners' accounts suggested that factors such as access to information, staff support, waiting times, opportunities for rest, and the overall organisation of care were more influential than the setting itself."

2. The data collection strategy differs substantially between participants and may have influenced the findings.

Thank you for pointing out the need for clarification. All participants completed the same two questionnaires. In addition, participants at the largest study site were asked an additional set of free-text questions in the postpartum questionnaire. We have clarified this in the Methods and Results. Although this may have increased the richness of data from that site, all responses were analysed together and experiences from both questionnaire formats contributed to the categories.
Page 8, line 181-188: "This study was based on qualitative inductive content analysis of written free-text answers collected through two electronic questionnaires administered within the OPTION trial (14). All partners received study-specific questionnaires administered at two timepoints; at inclusion before randomization and three months postpartum. The questionnaires included both fixed-response items and opportunities to provide free-text comments regarding experiences of overall experiences of the IOL process (Table 1)(14). The present study is based on the free-text responses. In a sub study at the largest site, participating partners were asked an additional set of free-text questions as part of the three months postpartum questionnaire (Table 1). "

3. Baseline and postpartum responses represent conceptually different types of data.

We agree that the responses collected at the two time points represent conceptually different perspectives. We consider this a strength of the study, as it captures the dynamic nature of the induction experience and reflects how perceptions may evolve over time. We acknowledge that the actual birth experience may influence participants' retrospective evaluation of induction. To improve clarity, we have revised the Results section to explicitly indicate whether findings are derived from baseline or postpartum responses and discuss the issue in the discussion:

Page 18, line 422-424: "As questionnaires were completed three months postpartum, participants may have reflected on the induction process considering the birth experience and outcomes. Consequently, some reported experiences may not exclusively relate to the induction process itself."

4. The 49% free-text response rate introduces important self-selection bias.

We apologise that this was not clear but 49% is not the response rate to the questionnaires, but reflects the percentage of those who left a free-text comment. Because of the qualitative design we aimed to seek variation within the responses not the number of responses. Please see our edit under Limitations:
Page 18, line 430-433: "As only 49% of participating partners provided free-text responses, the experiences reported may not fully reflect those of all partners participating in the OPTION trial. However, the use of questionnaire-based free-text responses enabled participation from a substantially larger and geographically more diverse sample than would have been feasible in an interview study."

5. Participant characteristics are insufficiently reported for assessing transferability.

Thank you for this suggestion. We agree that including additional partner-specific characteristics can improve the transferability of the findings. We have therefore added gender, randomization group and hospital type as further partner characteristics in Table 2a-b. In addition, we present maternal and birth-related characteristics, as we believe these factors may influence the overall induction experience and thereby support interpretation of the findings. We hope these additions improve the contextual understanding and transferability of the results. Please see Table 2a-b on page 25.

6. The distinction between characteristics of the partner and characteristics of the pregnant woman should be improved.

Thank you for pointing out the need for clarification. We edited the table accordingly. Please see Table 2a-b on page 25.

7. The potential influence of birth outcome and complications on the reported IOL experience deserves greater consideration.

We thank the reviewer for this important observation. We agree that partners' experiences of induction are likely influenced not only by the induction process itself but also by subsequent labour, birth outcomes, and complications. We have added information on the timepoint for answering to the citations in the Result section and expanded the Discussion to acknowledge that partners' retrospective accounts were collected three months postpartum and may reflect experiences related to labour progression, mode of birth, and birth outcome rather than the induction process alone:
Page 18, line 422-424: "As questionnaires were completed three months postpartum, participants may have reflected on the induction process considering the birth

experience and outcomes. Consequently, some reported experiences may not exclusively relate to the induction process itself."

8. The analytic procedure needs greater methodological detail.

We have expanded the description of the analytical procedure to provide greater transparency regarding the coding process, category development, management of disagreements, and the involvement of the authors throughout the analysis, please see Page 9, line 195-199: "The analysis followed the preparation, organisation, and reporting phases described by Elo and Kyngäs (16). During the preparation phase, all responses were read repeatedly to gain familiarity with the data. All free-text responses from the two questionnaire time points were analysed together as one dataset. Responses submitted in English were analysed alongside Swedish responses without translation during coding."
Page 9, line 205-208: "Through iterative discussion and constant comparison with the original responses, subcategories were further developed into broader categories representing the latent content of the data. Disagreements regarding interpretation were resolved through discussion until consensus was reached."

9. Researcher reflexivity is insufficient despite being indicated in the SRQR checklist.

We have elaborated the reflexivity statement describing the researchers' professional backgrounds, experience in maternity care, and how differing perspectives were used throughout the analytical process to strengthen interpretation and reduce the influence of individual preconceptions, please see
Page 9-10, line 210-214: "The authors consisted of midwives and obstetricians with experience of maternity and childbirth care and both in- and outpatient IOL care. The authors were aware that their clinical experience could influence data interpretation. To promote reflexivity, coding and interpretation were conducted independently before being discussed within the multidisciplinary research team."

10. The authors should clarify the methodological position regarding saturation or information sufficiency.

As this study was based on written free-text responses from a large cohort rather than sequential interviewing, data saturation was not used as a methodological criterion. Instead, we considered whether the dataset provided sufficient breadth and variation to address the study aim, please see
Page 18, line 417-420: "Due to the nature of data collection, recruitment was not guided by saturation. Instead, we considered the large number of participants, the variation in responses, and the breadth of experiences represented in the dataset to provide sufficient information to address the study aim."

11. Data richness should be described.

We thank the reviewer for this important observation. We have added a description of the richness and variation of the dataset in the Result section, please see
Page 10, line 223-225: "The free-text answers ranged from brief comments to detailed narratives describing experiences and reflections on the IOL process, providing both breadth and depth relevant to the study aim."

12. The wording regarding credibility and transferability is stronger than the available participant data support.

We have revised the wording regarding credibility and transferability to more cautiously reflect the strengths and limitations of a voluntary free-text dataset. The multicentre design and inclusion of partners with varying characteristics and experiences from both inpatient and outpatient IOL provided breadth to the dataset, please see
Page 18, line 414-421: "The inclusion of partners of both nulliparous and multiparous women, from all over Sweden, giving birth at university as well as minor hospitals, may support the relevance of the findings across similar maternity care contexts. Due to the nature of data collection, recruitment was not guided by saturation. Instead, we considered the large number of participants, the variation in responses, and the breadth of experiences represented in the dataset to provide sufficient information to address the study aim. However, transferability should be considered in light of the study population and design."

13. The findings related to outpatient IOL should be interpreted in the context of trial selection.

We thank the reviewer for this important observation. We agree that participation in the OPTION trial may have introduced selection considerations and have expanded the Discussion accordingly. An important aspect is that women – not necessarily their partners - needed to feel comfortable with the possibility of outpatient cervical ripening to participate in the trial. As noted above, several partners described that they themselves would have preferred an inpatient setting; however, they also expressed that the woman's preference was more important than their own preference regarding the setting. We have clarified this perspective in the Discussion to provide additional context for interpreting the findings, please see
Page 18, line 425-429: "When interpreting the findings, it should be noted that participants were recruited through the OPTION trial, and women who chose to participate may have been more favourably disposed towards outpatient IOL than the general population. However, our findings also included several accounts from partners who – themselves - preferred an inpatient setting. This suggests that perspectives on the preferred care setting varied among participants."

14. The manuscript should more clearly distinguish findings from clinical recommendations.

We have revised the Discussion to more clearly distinguish empirical findings from implications for practice, please see
Page 20, line 477-479: "Individualized information, structured care pathways, and supportive environments were described as helping partners feel better supported during IOL."

15. The term "pseudo-anonymous" requires clarification.

We have clarified what we mean by pseudo-anonymous responses, please see
Page 9, line 190-192: "Responses were pseudonymised, meaning that participants were assigned a study ID and direct identifiers were removed prior to analysis."

16. Minor spelling errors should be corrected.

We have corrected all spelling errors we found.

Reviewer: 2
Dr. Helen Cheyne, University of Stirling
Comments to the Author:
Thank you for inviting me to review this interesting article. The study aims to describe the experiences of partners of women undergoing labour induction, both in and outpatient, by analysing the free text data on online questionnaires. This is an interesting topic that is highly relevant currently as rates of labour induction rise in many countries. The experiences of partners are important but often overlooked and under researched.
Overall, this is a well written paper and enjoyable to read. I have a few specific comments and suggestions for minor changes. These mostly relate to the methods section which I felt could be more logically structured, with some more detail for clarity.

Thank you very much for taking the time to review our manuscript and providing valuable feedback.

Introduction
Line 127 For low-risk IOLs, outpatient IOL has been introduced in several countries. I suggest adding a sentence to explain outpatient IOL.

We thank the reviewer for this suggestion and have added a description of outpatient IOL to improve clarity for readers unfamiliar with this model of care, please see
Page 6, line 129-131: "Outpatient IOL typically involves initiation of IOL at the hospital, followed by the woman returning home during cervical ripening before being re-assessed or admitted when labour progresses or further intervention is required"

Despite partners often being the primary support person during the phase of cervical ripening – through most of this paper no distinction is made between cervical ripening

and the later phases of the IOL process. Do you intend this paper to make that distinction? This may be important as you also have a focus on outpatient – which generally relates only to the process of cervical ripening. If so, I think you need to thread the differences between cervical ripening and IOL throughout.

We agree that outpatient induction is primarily related to the cervical ripening phase, whereas the overall induction experience extends through subsequent stages of induction, labour and birth. The aim of the present study was to explore partners' experiences of the entire induction process rather than to distinguish between specific phases which was not supported by the nature of data collection. We have also highlighted this consideration in the Discussion as a potential influence on the findings. Page 18, line 422-424: "As questionnaires were completed three months postpartum, participants may have reflected on the IOL process considering the birth experience and outcomes. Consequently, some reported experiences may not exclusively relate to the IOL process itself"

The first two sentences of the concluding paragraph in the introduction repeat what is written in the paragraph above, I suggest removing the repetition.

We thank the reviewer for this important observation and have edited the text according to your suggestion.

Methods

Design – the sentence justifying the design doesn't really explain the design. I suggest adding an additional sentence in this section to clarify that this was a qualitative analysis of free text responses within online questionnaires. The current sentence justifying the method – as required by the checklist – is justifying content analysis not the questionnaire / free text comments approach that was actually the design of the study. I think this could be clearer.

We have edited the manuscript according to your suggestion, please see
Page 7 line 152-153: "This study was based on qualitative inductive content analysis of written free-text answers collected through two electronic questionnaires administered within the OPTION trial"

Line 155 onwards describing the OPTIONS Trial. The sentence Partners to participating women were invited to join the questionnaire part of the study. This does not fit well here. It does not give enough information about the partners participation, and it breaks up the description of the trial. I suggest completing the description of the Trial and then go on to describe the questionnaires more fully.

We thank the reviewer for this suggestion and moved the section on partner inclusion to Recruitment and Participation, please see
Page: 8, line 174-178:" Partners of women participating in OPTION were invited to join the questionnaire part of the study at the time of the woman's inclusion in the trial.

Partners participating in OPTION between 1 December 2021 and 24 August 2024 were eligible for the present study. All participating partners provided written informed consent before completing the first questionnaire."

Line 162 – you say - women were 163 randomized to continue IOL at the hospital, or to return home until the active phase of birth started – at few sentences later you say they were scheduled to return after 24 hours for assessment.

We thank the reviewer for pointing out the need for clarification, please see
Page 8, line 167-170: "All women in the outpatient group had a planned appointment with new cervix examination, foetal monitoring and plan for continued IOL care at the hospital after 24 hours. If there was need for further cervical ripening, the woman and her partner could return home for another 24 hours before final admission to the hospital."

Following completion of the description of the OPTIONS Trial, I suggest you go on to provide more detail on the questionnaires. For example, were the questionnaires just for partners or were they for the women but with some questions for partners? What were the questionnaires about?

We agree that the original manuscript did not provide sufficient information about the questionnaires. We have therefore expanded the Methods section to clarify that partners completed separate questionnaires as part of the OPTION trial. We have also included additional details regarding the questionnaire content, timing of data collection, and the free-text questions that formed the basis of the present analysis, please see
Page 8, line 181-188: "The study is based on free-text answers from electronic questionnaires completed by partners participating in the OPTION trial (14). All partners received study-specific questionnaires administered at two timepoints; at inclusion before randomization and three months postpartum. The questionnaires included both fixed-response items and opportunities to provide free-text comments regarding experiences of overall experiences of the IOL process (Table 1)(14). The present study is based on the free-text responses. In a sub study at the largest site, participating partners were asked an additional set of free-text questions as part of the three months postpartum questionnaire (Table 1)."

Recruitment – what was the procedure for recruitment of the partners and how did they provide written consent – for example, was their consent gained at the start of the electronic questionnaire or were they recruited at the same time as the women were initially recruited to the trial?

We have expanded the Recruitment and Participants section to describe how partners were recruited through the OPTION trial and how written informed consent was obtained prior to participation in the questionnaire study, please see
Page: 8, line 174-178:" Partners of women participating in OPTION were invited to join the questionnaire part of the study at the time of the woman's inclusion in the trial.

Partners participating in OPTION between 1 December 2021 and 24 August 2024 were eligible for the present study. All participating partners provided written informed consent before completing the first questionnaire."

Data collection – why were some partners given additional free text questions? Can you clarify if there were two or three questionnaires? The abstract says two but the first sentence in the Data Collection section suggests three – inclusion, randomisation and postpartum.

We thank the reviewer for identifying this lack of clarity. We have revised the Data Collection section to provide a more detailed description of the questionnaires, their timing, and their content. We have also clarified the reason why participants at the largest study site received additional free-text questions, please see under Data collection,
Page 8, line 182-188: "Partners received study-specific questionnaires administered at two timepoints; at inclusion before randomization and three months postpartum. The questionnaires included both fixed-response items and opportunities to provide free-text comments regarding experiences of overall experiences of the IOL process (Table 1)(14). The present study is based on the free-text responses. In a sub study at the largest site, participating partners were asked an additional set of free-text questions as part of the three months postpartum questionnaire (Table 1)."

Data analysis
This section is quite well described, please clarify if data for all questionnaire time points were analysed as one data set? If so, I wondered about the usefulness of having two or three questionnaires. Were any questionnaires returned in English and if so, how was data translation handled.

We thank the reviewer for this suggestion. We have clarified that all free-text responses, regardless of questionnaire time point, were analysed as a single dataset. As the aim was to describe partners' experiences of induction of labour rather than to investigate changes over time, we considered a combined analysis to be the most appropriate approach. However, we also consider the inclusion of responses from different time points to be a strength, as it captures both expectations prior to induction and experiences throughout the induction process. Please see
Page 9, line 196-198: "During the preparation phase, all responses were read repeatedly to gain familiarity with the data. All free-text responses from the two questionnaire time points were analysed together as one dataset."
Page 18, line 422-424: "As questionnaires were completed three months postpartum, participants may have reflected on the IOL process considering the birth experience and outcomes. Consequently, some reported experiences may not exclusively relate to the IOL process itself"

In addition, we have clarified the handling of language during the analysis. Responses submitted in English were analysed in their original language see

Page 9, line 198-199: "Responses submitted in English were analysed alongside Swedish responses without translation during coding"

How many of the responses were from partners in the home IOL arm of the study?

In the outpatient IOL arm of the study 252 (51.7%) partners answered any of the free-text questions. We have added the information to table 2, please see page 25.

Is there any more information available on the participants characteristics? for example, were all male? You claim that a strength of the study is the variation in participants but don't include much information to back that up.

We thank the reviewer for this comment. We agree that additional participant characteristics may help readers assess the variation represented in the dataset. We have therefore expanded the participant description and included additional available demographic characteristics in Table 2, please see page 25.

Findings

The findings are generally well written and interesting. I liked the summary of findings at the start but then going into theme one it seems a little repetitive with the first paragraph just repeating the summary above.

We agree that there was some repetition between the overall summary of findings and the introduction to the first category. We have therefore revised the text to reduce redundancy and improve the flow of the Results section, please see
Page 11, line 246-247: "Partners entered the IOL process with different expectations, previous experiences, and needs for reassurance, which shaped how they experienced IOL."

Is it possible to add some identifiers to the quotes? At present most of the quotes could have come from just one or two partners. Did most of the data come from the larger unit where more questions were asked?

We thank the reviewer for this suggestion. To improve transparency and illustrate the variation within the dataset, we have added identifiers to the quotations, including parity (nulliparous/multiparous) and questionnaire time point, while preserving participant anonymity.

Discussion

You say - Variation in participant characteristics and hospital settings strengthens the credibility and potential transferability of the findings. Yet you only include age and attendance at classes for partner variation and no data on hospital variation.

We thank the reviewer for this important observation. We have added additional characteristics, including hospital size, to Table 2 and revised the Discussion to use more cautious and nuanced wording when interpreting the findings. Please see Page 18, line 414-421: "The inclusion of partners of both nulliparous and multiparous women, from all over Sweden, giving birth at university as well as minor hospitals, may support the relevance of the findings across similar maternity care contexts. Due to the nature of data collection, recruitment was not guided by saturation. Instead, we considered the large number of participants, the variation in responses, and the breadth of experiences represented in the dataset to provide sufficient information to address the study aim. However, transferability should be considered in light of the study population and design."

VERSION 2 - REVIEW

**Reviewer**	**1**
**Name**	**Karataşlı, Volkan**
**Affiliation**	**Balikesir Atat□□rk City Hospital**
**Date**	**06-Oct-2026**
**COI**	

Thanks the authors for the corrections.
